# Effect of Single Amino Acid Substitutions by Asn and Gln on Aggregation Properties of Bence-Jones Protein BIF

**DOI:** 10.3390/ijms20205197

**Published:** 2019-10-20

**Authors:** Maria Timchenko, Azat Abdullatypov, Hiroshi Kihara, Alexander Timchenko

**Affiliations:** 1 Laboratory of NMR of Biosystems, Institute of Theoretical and Experimental Biophysics RAS, Pushchino 142290, Russia; 2 Laboratory of Biotechnology and Physiology of Phototrophic Organisms, Institute of Basic Biological Problems RAS—a separate subdivision of PSCBR RAS (IBBP RAS), Pushchino 142290, Russia; azatik888@yandex.ru; 3 Himeji-Hinomoto College, 890 Koro, Kodera-cho, Himeji 679–2151, Japan; kiharah1234@gmail.com; 4 Laboratory of Protein Physics, Institute of Protein Research RAS, Pushchino 142290, Russia; atim@vega.protres.ru

**Keywords:** Bence-Jones proteins, myeloma, aggregation, atomic force microscopy

## Abstract

The nature of renal amyloidosis involving Bence-Jones proteins in multiple myeloma is still unclear. The development of amyloidosis in neurodegenerative diseases is often associated with a high content of asparagine and glutamine residues in proteins forming amyloid deposits. To estimate the influence of Asn and Gln residues on the aggregation of Bence-Jones protein BIF, we obtained recombinant BIF and its mutants with the substitution of Tyr187→Asn (Y187N) in α-helix of C_L_ domain, Lys170→Asn (K170N) and Ser157→Gln (S157Q) in C_L_ domain loops, Arg109→Asn in V_L_-C_L_ linker (R109N) and Asp29→Gln in V_L_ domain loop (D29Q). The morphology of protein aggregates was studied at pH corresponding to the conditions in bloodstream (pH 7.2), distal (pH 6.5) and proximal renal tubules (pH 4.5) by atomic force microscopy (AFM) and small-angle X-ray scattering (SAXS). The Lys170→Asn replacement almost completely inhibits amyloidogenic activity. The Y187N forms fibril-like aggregates at all pH values. The Arg109→Asn replacement resulted in formation of fibril-like structures at pH 7.2 and 6.5 while the substitutions by Gln provoked formation of those structures only at pH 7.2. Therefore, the amyloidogenic properties are highly dependent on the location of Asn or Gln.

## 1. Introduction

Multiple myeloma nephropathy occurs due to the formation of different aggregates by immunoglobulin light chains (Bence-Jones protein) in kidneys of patients with multiple myeloma (malignant bone marrow disease), leading to lethal outcome. The mechanism of initiation of the fibrillation process is not clear. Previously, it was assumed that the key role in fibril formation belongs to the variable domains of immunoglobulin light chains due to somatic mutations in their structure. However, Solomon et al. have reported about the first case of amyloid light-chain (AL) amyloidosis in a patient with κ Bence-Jones proteinuria, when fibrillar deposits were not formed of V_L_-domain but, rather, consisted almost entirely of C_L_, containing a germLine encoded Ser→Asn amino acid substitution at position 177 [1]. 

In previous studies it was found that high content of Asn and Gln in proteins play a crucial role in aggregation associated with the development of neurodegenerative diseases [2]. It is known that the presence of multiple Asn and Gln repeats in protein sequences is often a key factor for amyloid formation. A large number of Gln repeats (encoded by CAG codons) is responsible for the occurrence of a number of neurodegenerative diseases, including Huntington disease [3]. It was found that in a case of the presence of poly-Gln regions in proteins the formation of protofibrils or intermediates at the beginning of the aggregation process is more destructive for cells than the formation of large fibrillar aggregates [4,5].

The reason of amyloidosis for Bence-Jones proteins is still unclear. The Bence-Jones protein BIF discovered by Solomon [1] has Asn and Gln rich sequence. The substitution Ser177→Asn in Bence-Jones protein BIF [1] is most likely essential for the initiation of amyloid formation process due to the appearance of additional Asn in protein as in the case of neurodegenerative diseases. Previously, we have shown that the reverse mutation, Asn→Ser177, completely suppressed the amyloidogenic properties of BIF [6]. To check the influence of Asn and Gln on aggregation process, we obtained recombinant protein BIF and its analogues with the substitution of one of the amino acids in several structural protein motifs for Asn: Tyr187 (Y187N protein) in C_L_ domain α-helix, Lys170 (K170N protein) in C_L_ domain loop and Arg109 in V_L_-C_L_ linker (R109N protein). Besides, additional Gln residues were introduced into the loops of the V_L_ domain (D29Q protein, Asp29→Gln) and C_L_ domain (S157Q protein, Ser157→Gln). According to the most recent data, the introduction of such residues into disordered regions of proteins can stimulate the formation of fibrils in neurodegenerative diseases; hence, the appearance of these substitutions in flexible parts of BIF may increase its amyloidogenic potential [7]. Here we present the study of influence of replacement of certain amino acids in BIF sequence with Asn and Gln on its aggregation process and morphology of obtained aggregates under different ionic conditions simulating native environment of Bence-Jones protein in kidneys.

## 2. Results

### 2.1. Analysis of Morphology of Aggregates 

**Figure 1:**
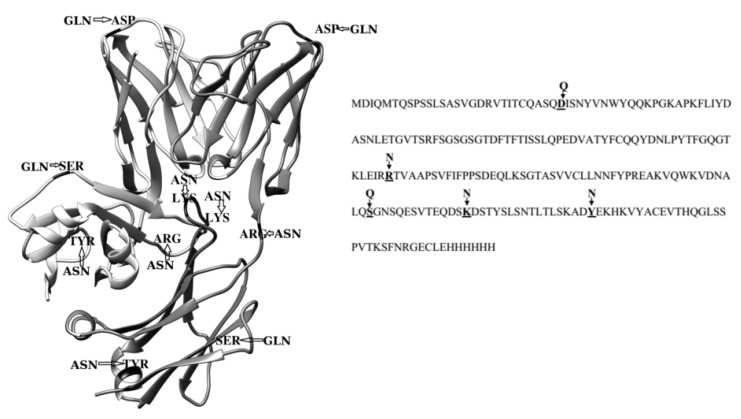
The scheme of locations of point mutations in amino acids sequence of BIF protein and in its C_L_ and V_L_ domains (the structure of immunoglobulin kappa light-chain dimer (Bence-Jones protein) DEL (PDB ID: 1B6D) was used as a model [10]). The picture was built using Chimera 1.13.1 (UCSF, San Francisco, CA, USA).

To study the influence of amino acid replacement with Asn residues on the aggregation of Bence-Jones protein BIF, the following mutant analogues were created: (1) Lys170 in C_L_ domain loop was substituted by Asn (K170N protein); (2) Tyr187 was replaced with Asn in C_L_ domain α-helix (Y187N protein); (3) Arg109 was replaced with Asn in the linker between V_L_ and C_L_ domains (R109N protein). The additional Gln residues were introduced by Asp29→Gln substitution in V_L_ domain (D29Q protein) and Ser157→Gln in C_L_ domain (S157Q protein) (Figure 1). 

The choice of mutations D29Q, S157Q and K170N was based on the empirical rule that the amyloid-prone region has to be located in a structurally disordered region, allowing its self-assembly without the necessity of conformational unfolding [7]. The Arg109 is important for the stabilization of C_L_ domain [8,9]. Therefore, the substitution of Arg109 by Asn could be crucial and could facilitate the formation of fibrils. Y187N mutation was chosen as an example of substitution in structurally ordered region (alpha-helix). According to Sabate et al., the occurrence of tyrosine in prion-forming domains (PFDs) is moderately elevated, whereas asparagine is the most overrepresented residue in PFDs [7]. Hence, such a mutation could increase amyloidogenic properties of BIF in case if the requirement for its location in structurally disordered region is not absolutely strict.

The numbering is shifted by one amino acid relative to the Kabat numbering system [11] due to the inclusion of the first methionine in the recombinant protein. The comparison of aggregation properties and morphology of protein aggregates under different conditions was performed using atomic force microscopy (AFM). Three buffer systems were used in this study: 1–50 mM phosphate buffer with 0.10 M NaCl, pH 7.2; 2–50 mM phosphate buffer with 0.4 M NaCl and 0.4 M urea, pH 6.5; 3–30 mM acetate buffer with 0.245 M NaCl, pH 4.5. These buffer systems were chosen to reflect environments affecting immunoglobulin light chains within nephron [12]. Buffer 1 represents conditions expected during the transport of protein in bloodstream and its filtration in the glomerulus. Buffer 2 contains urea and salt to emulate the microenvironment of the distal tubule. The salt concentration reflects the condition of partial dehydration, which results in the development of renal pathologies involving Bence-Jones proteins, and the chosen urea concentration is considerably less than that typically required to solubilize proteins. Buffer 3 with low pH simulates the conditions in the renal proximal tubule (the place of light chain catabolism as well as urine acidification). The acidification is considered as a contributing factor to the nephrotoxicity of Bence-Jones proteins. 

**Figure 2:**
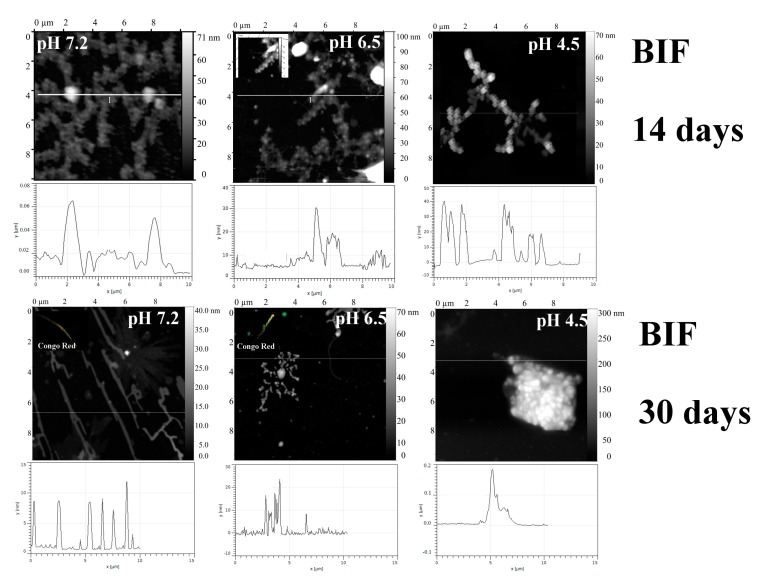
The morphology of BIF aggregates at pH 7.2, 6.5 and 4.5 after 14 days and 30 days of incubation at 37 °C (AFM images obtained in tapping mode). Congo Red birefringence is shown. The data on top were taken from [6]; the authors (Timchenko M.A., Timchenko A.A.) retain the right to use the images in other works according to copyright transfer agreement.

As previously shown [6], BIF exhibited amyloidogenic properties at pH 7.2 and 6.5, while at pH 4.5 it did not form fibrils (Figure 2). 

**Figure 3:**
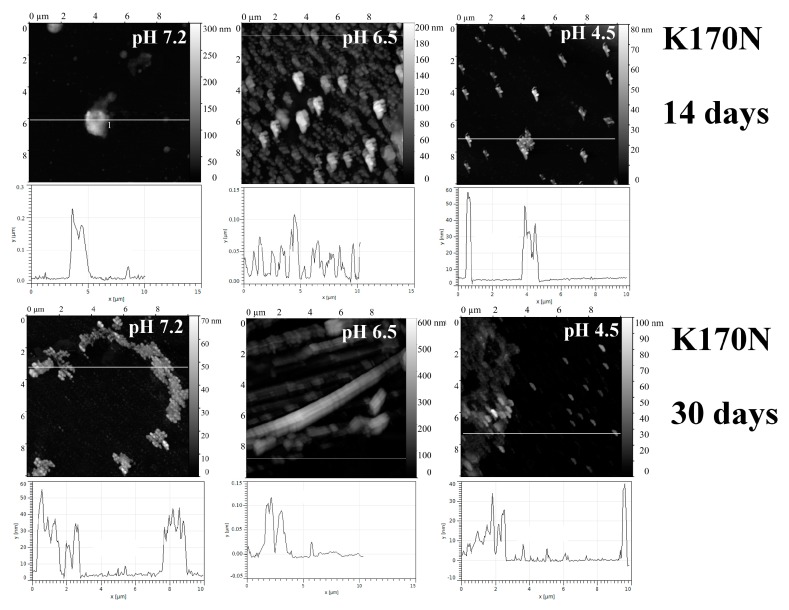
The morphology of K170N aggregates at pH 7.2, 6.5 and 4.5 after 14 days and 30 days of incubation at 37 °C (AFM images obtained in tapping mode).

The replacement Lys170→Asn in C_L_ domain loop (K170N) significantly changed the amyloidogenic activity of BIF. In contrast to BIF protein, K170N formed amorphous aggregates consisting of small oligomeric structures at all studied pH. Rod-shaped structures were found only at pH 6.5 after a month of incubation at 37 °C (Figure 3).

**Figure 4:**
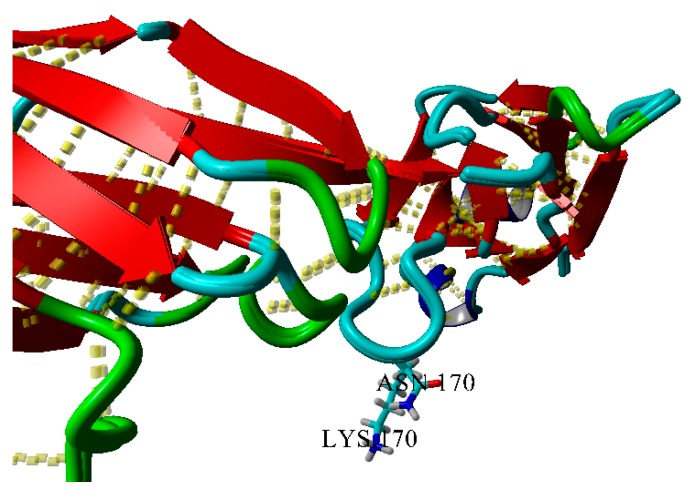
Superposition of wild-type and K170N BIF variants. 3D dashed lines show hydrogen bonds.

Figure 4 shows superposition of wild-type protein BIF and mutant K170N for comparison and analysis of the contribution of lysine to the internal and external interactions of the protein. The K170N mutation appears quite contradictive. On one hand, the surface of the protein becomes less prominent. On the other hand, lysine residue is a worse hydrogen-bonding partner than asparagine, since the latter can form two acceptor and two donor hydrogen bonds. This contradiction is realized in non-amyloidogenic or even anti-amyloidogenic nature of this mutation, so that the surface effect could overwhelm the enhanced hydrogen bonding ability. In other words, hydrogen bonding is most likely sterically impeded in case of this mutation.

**Figure 5:**
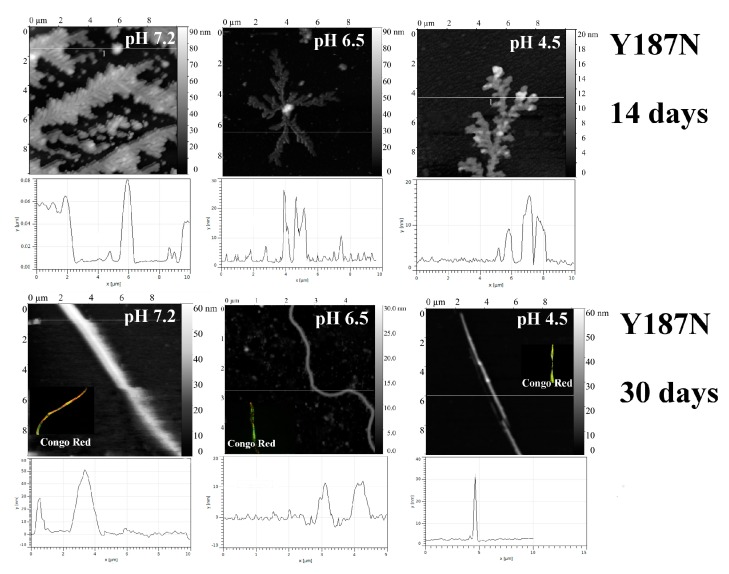
The morphology of Y187N aggregates at pH 7.2, 6.5 and 4.5 after 14 days and 30 days of incubation at 37 °C (AFM images obtained in tapping mode). Congo Red birefringence is shown.

The introduction of Asn into a short α-helix in C_L_ domain (Y187N protein) resulted in the appearance of fibril-like structures at all studied pH values (Figure 5). At pH 7.2, Y187N formed rod-like structures, most likely due to the association of several fibril-like structures.

This mutation had the most dramatic effect on amyloidogenic properties of BIF protein. Its mechanism is complicated and cooperative. 

**Figure 6:**
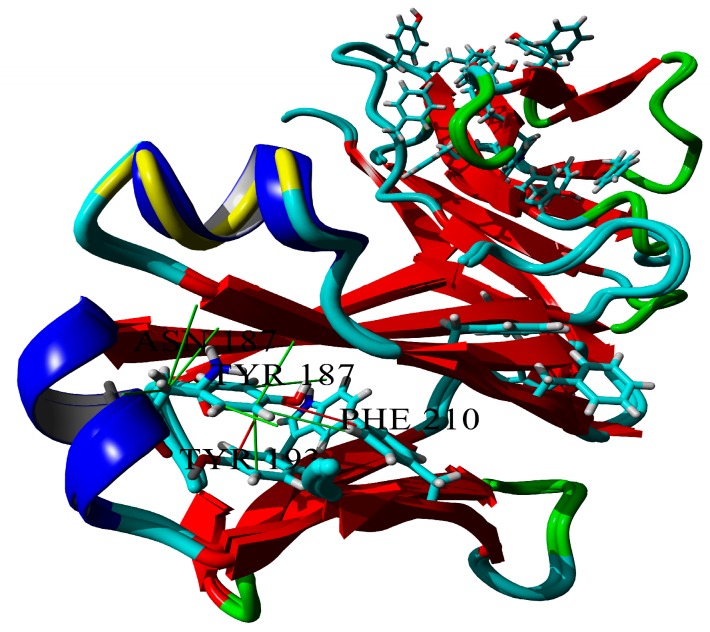
Hydrophobic core formed by Y187. Hydrophobic contacts are shown in green, π-π interactions are shown in red (cutoff = 5 Å). π-π partners of Y187 are labeled, and tryptophan residue, which is “a partner of a partner”, is shown. Superposition of wild-type and Y187N BIF variants.

The tyrosine residue, Y187, plays a central role in hydrophobic core formation. It forms at least two cation-π interactions (stacking) and a large number of hydrophobic interactions, most of which vanish when substituting it to asparagine (Figure 6; only three of the eight hydrophobic contacts can be conserved). 

**Figure 7:**
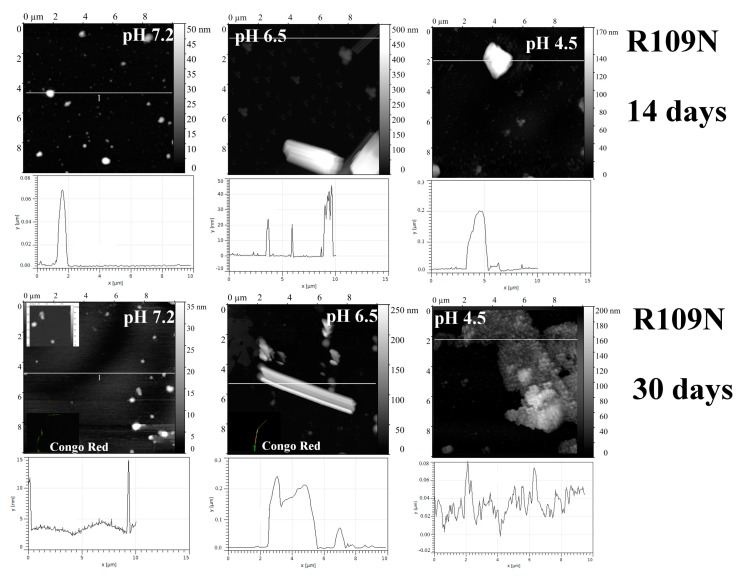
The morphology of R109N aggregates at pH 7.2, 6.5 and 4.5 after 14 days and 30 days of incubation at 37 °C (AFM images obtained in tapping mode). Congo Red birefringence is shown.

The Arg109→Asn replacement in interdomain linker resulted in significant formation of fibril-like structures at pH 6.5 (Figure 7). The fibrils were also detected at pH 7.2 after a month of incubation but the mechanism of its formation presumably differs from that for BIF (Figure 2 and Figure 7, respectively). At pH 4.5, protein R109N aggregates with formation of large amorphous structures (Figure 7). 

**Figure 8:**
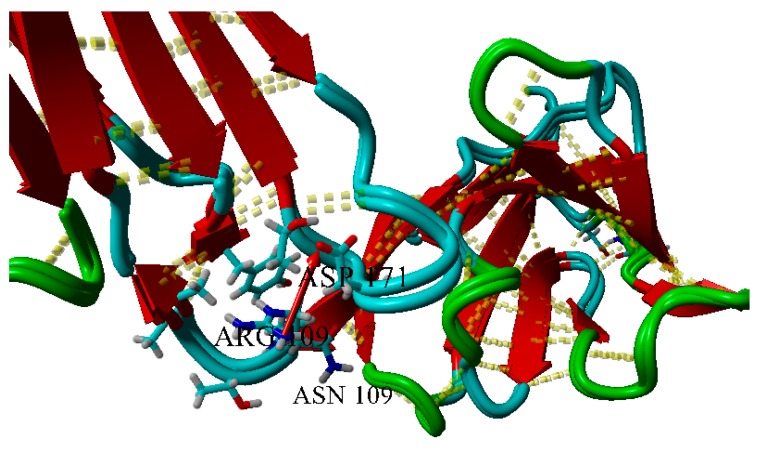
Superposition of wild-type and R109N variants of BIF. The red arrow shows a weak (d = 5.969 Å) ionic pair. 3D dashed lines show hydrogen bonds.

Arginine residue can form a weak ionic pair with D171 residue; in the mutant R109N this interaction is broken (Figure 8).

**Figure 9:**
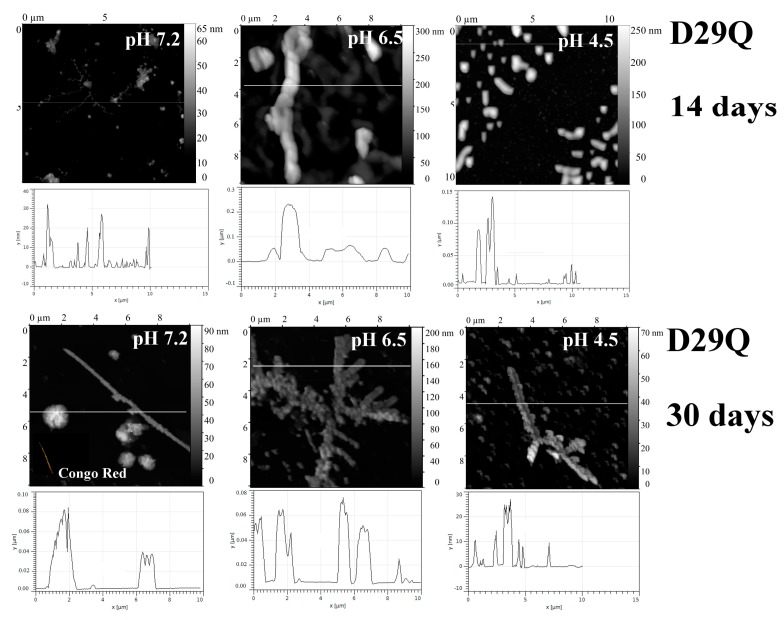
The morphology of D29Q aggregates at pH 7.2, 6.5 and 4.5 after 14 days and 30 days of incubation at 37 °C (AFM images obtained in tapping mode). Congo Red birefringence is shown.

**Figure 10:**
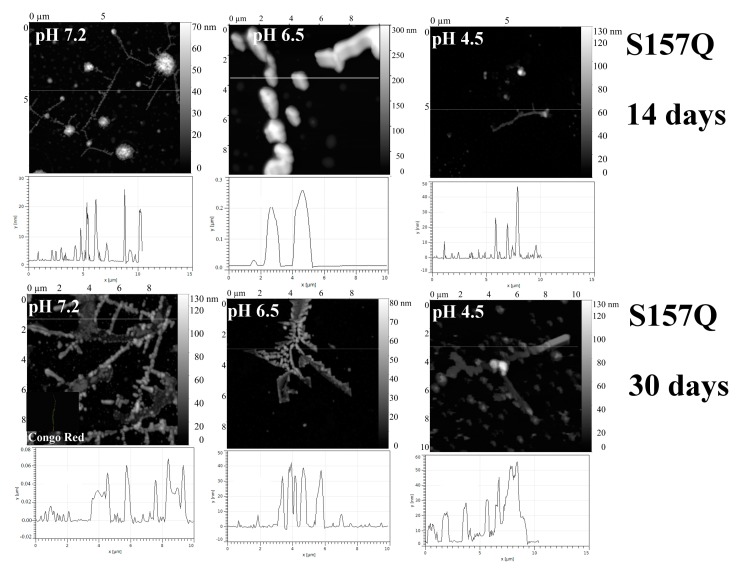
The morphology of S157Q aggregates at pH 7.2, 6.5 and 4.5 after 14 days and 30 days of incubation at 37 °C (AFM images obtained in tapping mode). Congo Red birefringence is shown.

Additional Gln residues introduced into the loops of the V_L_ domain (D29Q protein) and C_L_ domain (S157Q protein) stimulate formation of fibril-like structures at neutral pH, while at pH 6.5 and 4.5 amyloids were not found (Figure 9 and Figure 10, correspondingly).

**Figure 11:**
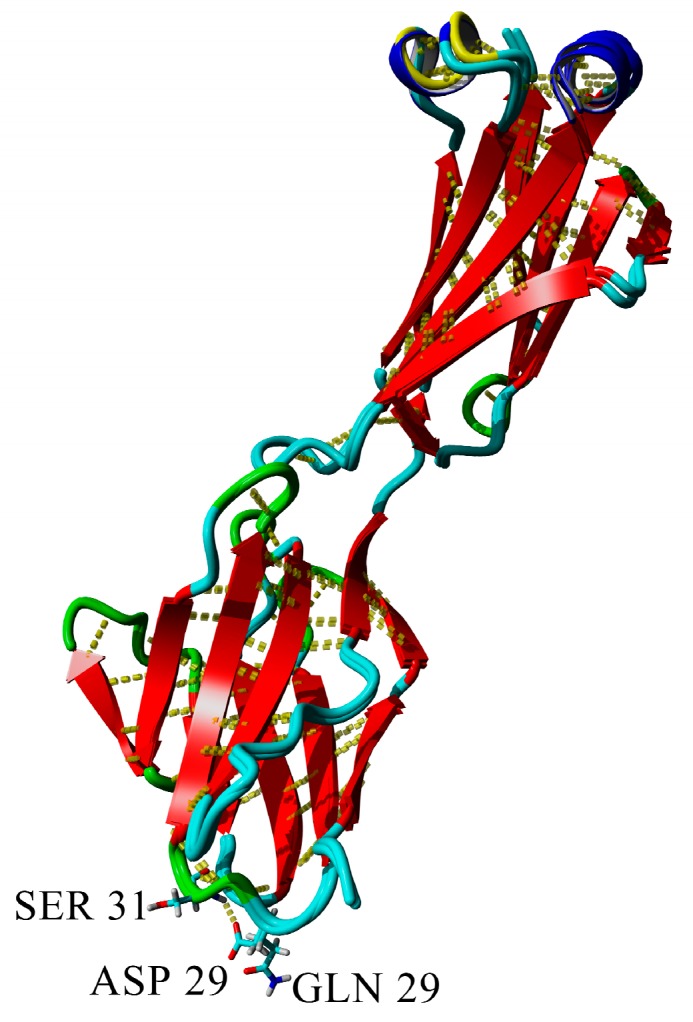
Superposition of wild-type and D29Q variants of BIF. Difference between wild-type of BIF and D29Q mutant: Wild-type forms a hydrogen bond with S31, whereas in the mutant the hydrogen bond is lost.

**Figure 12:**
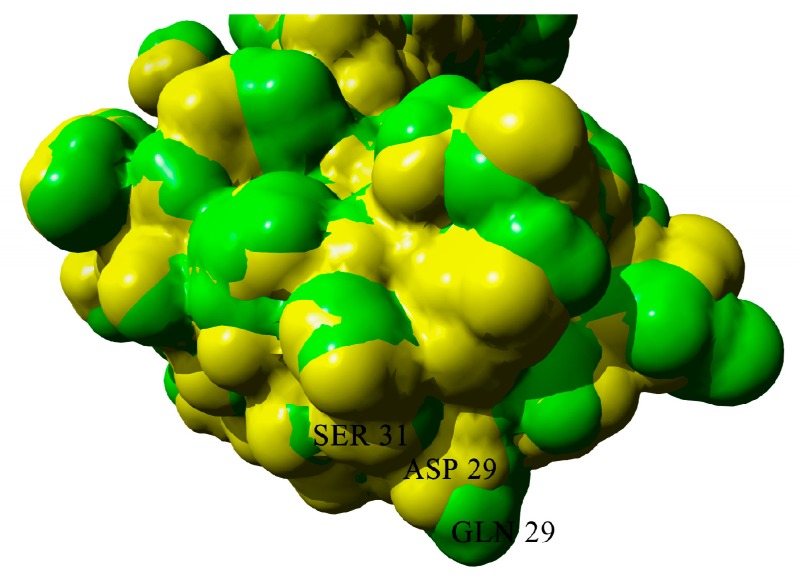
Solvent-accessible surfaces of wild-type BIF (yellow) and D29Q mutant (green).

The D29 residue can form a hydrogen bond with S31. The substitution of this residue to glutamine leads to the elongation of the side chain and reduction of the probability of hydrogen bond formation, because it can be formed only when the CO-group of the side-chain is oriented towards the NH group of serine main chain (Figure 11). Moreover, the solvent-accessible surface of the mutant is more prominent than that of wild-type protein (Figure 12)

**Figure 13:**
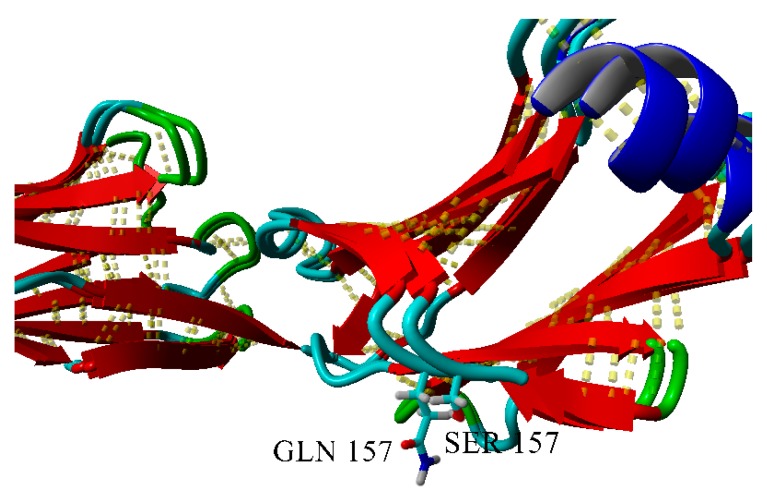
Superposition of wild-type and S157Q variants of BIF.

**Figure 14:**
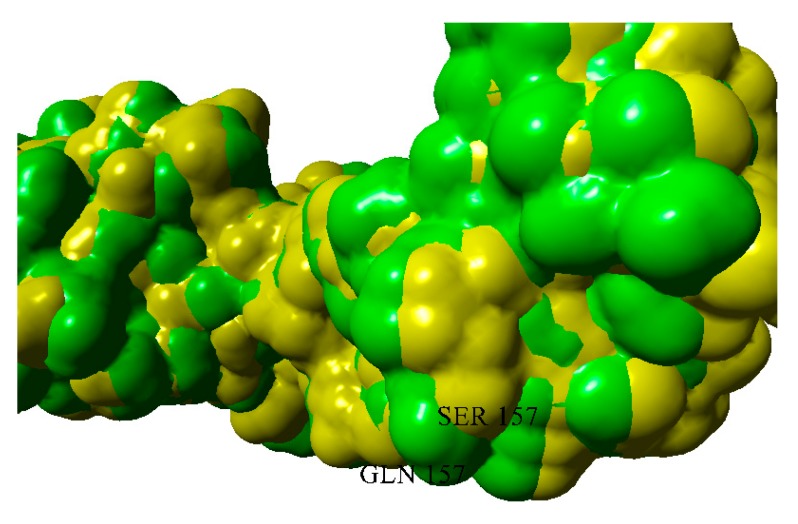
Solvent-accessible surfaces of wild-type BIF (yellow) and S157Q mutant (green).

S157Q mutation makes the local surface more prominent; moreover, it should be emphasized that glutamine residue is a good hydrogen bond donor for aggregation (Figure 13 and Figure 14).

**Figure 15:**
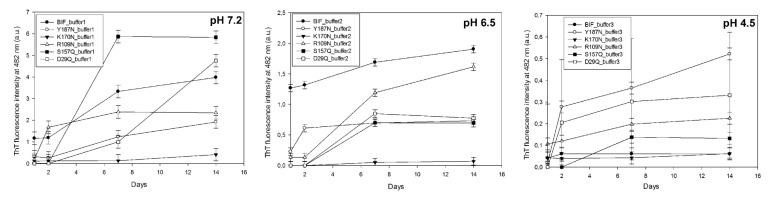
ThT fluorescence in presence of 1 μg/mL protein at different pH.

The presence of fibrils in addition to AFM observations was confirmed by analysis of birefringence of samples incubated for a month at 37 °C in polarized light after Congo red staining, by thioflavin T (ThT) (Figure 15) and Congo red (CR) spectroscopic assays and by analysis of resistance of samples to 2% SDS in gel-electrophoresis (Table A1).

In view of the obtained results, it can be assumed that the most crucial changes in aggregation properties of BIF were obtained by replacements of Lys 170 and Tyr 187 with Asn. The substitution Lys170→Asn was crucial for the amyloidogenic activity of BIF, whereas the substitution for Asn in C_L_ domain α-helix (Y187N protein) resulted in the formation of fibril-like structures at all studied pH values. Other substitutions had no such pronounced effect. The substitutions D29Q and S157Q slightly increased the formation of fibrils at pH 7.2 and did not reveal amyloidogenic properties at pH 6.5, whereas the replacement R109N resulted in rapid fibril growth at pH 6.5. Thus, the amyloidogenic properties are very sensitive to the site of substitution.

### 2.2. Small Angle X-Ray Scattering (SAXS) Data

**Figure 16:**
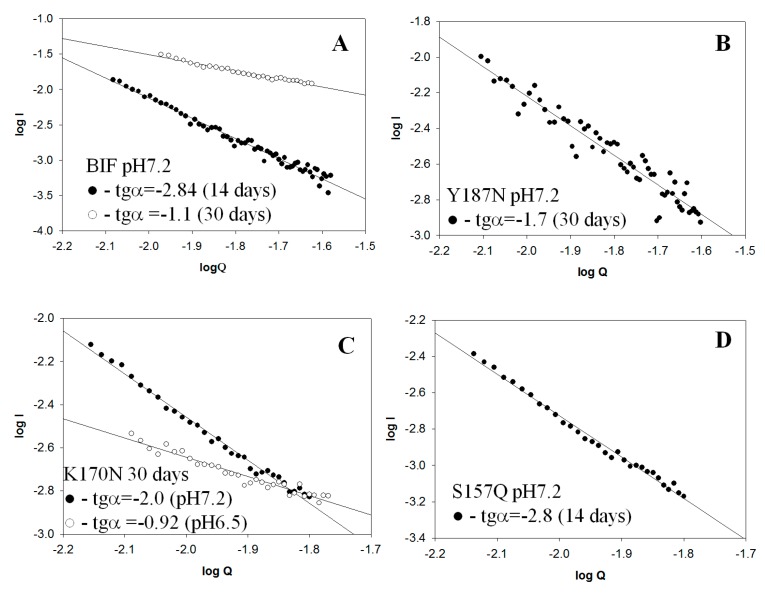
SAXS patterns (LogI vs. LogQ): (**A**) BIF at pH 7.2 after 14 and 30 days of incubation at 37 °C; (**B**) Y187N at pH 7.2 after 30 days of incubation at 37 °C; (**C**) K170N at pH 7.2 and 6.5 after 30 days of incubation at 37 °C; (**D**) S157Q at pH 7.2 after 14 days of incubation at 37 °C.

The tendency of proteins to aggregation complicated the performance of SAXS experiments; however, some information on their conformation in aggregates can be obtained, as was demonstrated by us for myoglobin [13]. We investigated conformation of oligomers plotting dependence logI-logQ where; I—intensity, Q—module of scattering vector. As known [14] for globular structures, slope of regression line (tgα) is −4, for flat ones −2 and for linear ones −1. The results were obtained for BIF protein at pH 7.2 after 14 days its incubation at 37 °C [6] and after a month of incubation (Figure 16A). Previously it was shown [6] that after two weeks of incubation, BIF formed elongated branched structures (tgα = −2.84), further incubation resulted in linear structures formation (tgα = −1.1). The results for Y187N were obtained for samples incubated for 30 days at pH 7.2. It was found that tgα for Y187N is −1.7, which points at the presence of mixed associates with the predominance of rod-like structures (Figure 16B). The shape of K170N was obtained for samples incubated for 30 days at pH 7.2 and 6.5. At pH 7.2, the protein had a predominantly flat elongated shape (tgα = −2.0) while at pH 6.5, the shape is closer to linear (tgα = −0.92) (Figure 16C). For S157Q incubated for two weeks at pH 7.2 tgα was −2.8 (Figure 16D), which indicates the presence of branched flat associates. The obtained data are in agreement with AFM results. Measurements in other buffer conditions for aforementioned proteins and for mutants R109N, D29Q did not allow to obtain scattering curves with acceptable accuracy due to low protein concentration because of precipitation.

### 2.3. Dimerization of BIF Molecules: Role of Surface Residue Substitutions

Among the mutations listed above, three are most likely acting via altering oligomerization (S157Q, D29Q and K170N). Since Bence-Jones protein is a dimer, its structure was used for reconstruction of dimeric proteins. 

However, all the three selected residues do not participate in dimerization of the protein. 

**Figure 17:**
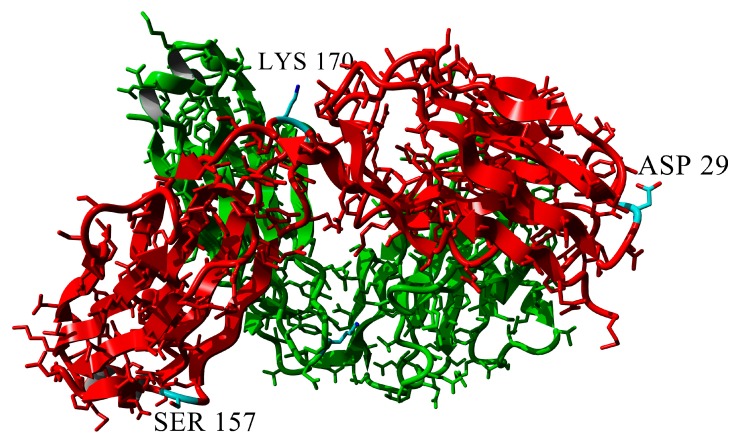
Location of three mutations affecting the kinetics of amyloidogenesis. Wild-type residues are shown, and they are labeled in one subunit of the dimer (top, red).

Even K170N mutation is not likely to alter dimerization of the native enzyme, since the 170th residue of the chain is located very far (distance more than 9Å) from the residues of the second molecule of a dimer. As for other mutations, they are located even farther (Figure 17).

## 3. Discussion

As it is known, fibrils and amorphous deposits of immunoglobulin light chains—Bence-Jones proteins play the key role upon development of renal nephropathy in multiple myeloma. The mechanism of amyloid nephropathy progression has not yet been fully understood. Generally, amyloid deposits in the kidneys of patients with multiple myeloma were due to oligomerization of the V_L_ domain of the immunoglobulin light chain [15,16,17,18,19]. It has been suggested that the presence of several key amino acids in the variable domain such as Asn, Asp, Gln, Pro, Ile promotes fibrillation [20,21]. However, Solomon et al. isolated the Bence-Jones protein BIF, whose amyloid deposits were represented mainly by the light chain’s C_L_-domain, and it was suggested that the amyloidogenicity of the protein is associated with the replacement of Ser177 by Asn in the amino acid sequence of the C_L_-domain, and this mutation is hereditary [1]. Previously, we have shown [6] that the reverse substitution of Asn177 for Ser in recombinant BIF protein resulted in suppression of amyloidogenic activity and inhibition of aggregation process. Moreover, Nowak et al. [21] also revealed that the replacement of Arg61 with Asn in the variable domain of another Bence-Jones protein REI provoked the formation of amyloid fibrils. Therefore, the introduction of only one Asn into the protein sequence significantly influenced its amyloidogenic properties. It is known that the key factor promoting amyloid formation in neurodegenerative diseases is the high content of Asn and Gln residues in protein [2]. Such studies were not carried out for Bence-Jones proteins. It is possible that the appearance of asparagine and glutamine residues due to various mutations leads to the development of renal amyloidosis in multiple myeloma. 

To elucidate how the high content of Asn and Gln residues influences on the process of fibrillogenesis, we obtained recombinant protein BIF and its analogues with the substitution Tyr187→Asn in the short α-helix of C_L_ domain of BIF (Y187N), Lys170→Asn in one of the C_L_ loops (K170N), Arg109→Asn in V_L_-C_L_ linker (R109N). Previously, Nokwe et al. [8] have shown that the Arg109 residue explains the stabilizing effect on the V_L_ domain, and for the C_L_ domain the interaction of N-terminal loop residues with Arg109, which is important for the integrity of the domain and its stabilization. Additional Gln residues were introduced into the loops of the V_L_ domain (D29Q protein) and C_L_ domain (S157Q protein), since according to recent data, the introduction of such residues into disordered regions of proteins can stimulate the formation of fibrils [7]. The morphology of aggregates was studied in three buffers reflecting nephron environments (pH 7.2, 6.5 and 4.5). The results are summarized in Table A1. 

It was shown that the tendency to aggregation increases for all proteins except K170N, in comparison with BIF protein after the replacement of corresponding amino acids in protein sequence by Asn and Gln. The introduction of Asn into interdomain linker (R109Nprotein) resulted in significant formation of fibril-like structures at pH 6.5 while additional Gln residues in loops of C_L_ and V_L_ domains (S157Q protein and D29Q protein) resulted in significant formation of those structures only at pH 7.2. At the same time, S157Q and D29Q did not form amyloid structures at other pH values, despite their high tendency to aggregate. The substitution of Lys170 for Asn (K170N protein) resulted in prevention of fibril formation at all studied pH. The formation of rod-like structures for K170N was observed only at pH 6.5 after a month of incubation. The introduction of Asn into the short α-helix (Y187N) resulted in the formation of fibril-like structures at all pH values.

Thus, the ability of mutant proteins of BIF to form amyloids depends on the location of mutation in protein structure. The appearance of Asn in interdomain linker and α-helix of the protein enhanced the formation of fibril-like structures. The substitutions for Gln residues in BIF stimulated the formation those structures under neutral conditions. Therefore, the replacement of amino acids in Bence-Jones proteins with Asn and Gln due to different mutations most likely plays an essential role in the development of renal amyloidosis, as well as in the case of amyloidosis in neurodegenerative diseases. However, due to the fact that the amyloidogenic properties of the Bence-Jones protein are very sensitive to the location of introduced mutations, further studies should be focused on the identification of the most sensitive regions for the substitutions and on revealing the mechanisms of involvement of Asn and Gln residues into fibrillogenesis. Understanding the process of the initialization of fibrillogenesis in multiple myeloma will help to develop the approach, enabling the prediction the renal amyloidosis progression for patients with multiple myeloma.

## 4. Materials and Methods 

### 4.1. Construction of the BIF-Expressing Plasmid pETBIF 

Amino acid sequence of BIF protein [1] was translated according to the standard genetic code into the coding nucleotide sequence with codon optimization for the expression in *Escherichia coli* The DNA fragment encoding this sequence was synthesized by polymerization reaction with overlapping oligonucleotides using the Velocity DNA polymerase (“Bioline”, Luckenwalde, Germany) and then amplified by the polymerase chain reaction (PCR). The obtained PCR fragment was cloned into NdeI and XhoI restriction sites into the pET23b expression vector (“Novagen”, Madison, WI, USA) under the control of T7 RNA-polymerase promotor. The presence of the insert was confirmed by the restriction and sequence analysis. At the C-terminus this construct contained the (His)_6_-tag for protein purification by affinity chromatography. Previously, the recombinant analogues of native BJP were studied in a number of works and they reliably reflected the structural peculiarities of native proteins [22,23].

### 4.2. Site-Directed Mutagenesis

The mutant analogues of BIF protein with substitution Tyr187→Asn (Y187N), Lys170→Asn (K170N), Arg109→Asn (R109N), Asp29→Gln (D29Q), Ser157→Gln (S157Q) were obtained by “Evrogen” company (“Evrogen”, Moscow, Russia). The accuracy of mutagenesis was confirmed by sequencing.

### 4.3. Expression and Purification of the Recombinant BIF Protein and Its Mutant Analogues 

Plasmids were transformed into *E. coli* cells (BL21(DE3)) by standard method [24]. Expression was performed in conical flasks with mixing in 1 liter of LB-medium [24], containing carbenicillin (0.1 g/liter) (“Bioline”, Luckenwalde, Germany). The cells were grown at 37 °C. Overnight culture was added at 1:100 dilution. Expression of gene of recombinant protein was induced by addition of isopropyl β-D-thiogalactopyranoside (IPTG) (“Bioline”, Germany) at a final concentration of 1 mM when the optical density of the culture reached 0.5 (λ = 550 nm). After 6 h of incubation of the culture at 37 °C the cells were pelleted by centrifugation at 3000 g for 15 min at 4 °C. The pellet of the cells was frozen and stored at −20 °C up to use.

Cells collected from 1 liter of culture were resuspended in 50 mL of NPI-10 buffer (50 mM NaH_2_PO_4_, 300 mM NaCl, 10 mM imidazole, pH 8.0), lysozyme (“Amresco”, Solon, OH, USA) was added up to a final concentration of 1 mg/mL, cells were mixed in ice for 30 min and disintegrated by sonication. The obtained lysate was centrifuged for 30 min at 10,000 *g* and at 4 °C, the supernatant was removed, the pellet containing inclusion bodies was resuspended in 20 mL of NPI-10 for washing and centrifuged for 30 min at 10,000× *g* and 4 °C, the supernatant was removed. The pellet was dissolved in 10 mL of NPI-10 buffer containing 6 M guanidine hydrochloride (“Amresco”, Solon, OH, USA), PMSF (phenylmethylsulfonyl fluoride) (“Amresco”, Solon, OH, USA) was added up to a final concentration of 1 mM and the suspension was sonicated. After sonication, Tween-20 detergent (“Amresco”, Solon, OH, USA) was added to the sample up to a final concentration of 2% and the suspension was mixed for 1 h in ice. The lysate was centrifuged for 30 min at 10,000 *g* and at 20 °C to remove the remaining insoluble fraction and transferred into clean eppendorf tubes. The sample was dialyzed against NPI-10 buffer, containing 6 M urea (“Amresco”, Solon, OH, USA), and loaded on Ni-NTA-Sepharose column (“Novagen”, Madison, WI, USA), equilibrated with NPI-10 buffer with 6 M urea. The column was washed with 10 volumes of NPI-20 buffer (50 mM NaH_2_PO_4_, 300 mM NaCl, 20 mM imidazole, pH 8.0) containing 6 M urea and the bound protein was eluted with one volume of NPI-250 buffer (50 mM NaH_2_PO_4_, 300 mM NaCl, 250 mM imidazole, pH 8.0) with 6 M urea. The presence of protein was confirmed by LaemmLi gel-electrophoresis [25].

### 4.4. Refolding of the Recombinant BIF Protein and Its Mutant Analogues 

The refolding of proteins was performed according to the procedure [26]. The protein-containing fractions collected after affinity chromatography were sequentially dialyzed for 12 h against NP phosphate buffer (50 mM NaH_2_PO_4_, 300 mM NaCl, pH 8.0), containing 6, 3 and 1 M urea. Insoluble aggregates of proteins precipitated and were separated by centrifugation at each stage of dialysis. The subsequent refolding was performed by controlled dialysis. NP buffer containing 1 M urea was replaced with 50 mM sodium-phosphate buffer, pH 7.2, without urea for 48 h (10% buffer volume per hour). After centrifugation the supernatant was divided into three fractions, which were dialyzed for 12 h against 50 mM sodium-phosphate buffer, pH 7.2 with 0.1 M NaCl, 50 mM sodium-phosphate buffer, pH 6.5 with 0.4 M NaCl and 0.4 M urea, 30 mM sodium-acetate buffer, pH 4.5, with 0.245 M NaCl, correspondingly. 

The obtained samples were filtered through the 0.22 µm filter (“Millipore”, Burlington, MA, USA). The yield of protein was 0.1 to 0.3 mg/mL. The presence of protein and its purity was confirmed by LaemmLi gel-electrophoresis [25].

### 4.5. Formation of Aggregates of BIF Protein and Its Mutant Analogues in Solution

The filtered samples of protein in the appropriate buffer were incubated at 37 °C for 14 days. Every 24 h, a sample of 50 µL was collected to analyze the self-association of proteins by atomic force microscopy (AFM) and other methods. At the first time point (one day), a sample of protein was taken, which was obtained immediately after dialysis in the appropriate buffer and protein filtration. 

### 4.6. The Thioflavin T (ThT) Assay

Ten µL of protein solution (0.1 mg/mL) was added to 1 mL of buffer solution 10 mM Na_2_HPO_4_, 150 mM NaCl, pH 7.0, containing Thioflavin T (0.016 mg/mL) (“Sigma-Aldrich”, Saint Louis, MS, USA. The fluorescence intensity of solution was measured with spectrofluorimeter “Cary Eclipse” (“Varian”, Sydney, Australia) at 482 nm, excitation at 440 nm [27]. From the obtained value, the fluorescence intensity of ThT in the absence of protein was subtracted (0.016 mg/mL)).

### 4.7. Congo Red Spectroscopic Assay

Ten µL of protein solution (0.1 mg/mL) was added to 1 mL of buffer solution 5 mM Na_2_HPO_4_, 150 mM NaCl, pH 7.4, containing Congo Red (“Sigma-Aldrich”, Saint Louis, MS, USA) (34.8 µg/mL). After incubation for 30 min at room temperature, the protein spectrum was recorded in the range 400–700 nm (quartz cell, 1 cm) using spectrophotometer UV-2401 (“Shimadzu”, Kyoto, Japan). From the obtained spectra the spectrum of Congo Red in the absence of protein was subtracted, a maximal spectral difference at 540 nm is indicative of amyloid fibrils [28].

### 4.8. Electrophoretic Analysis of Aggregates in Polyacrylamide Gel (PAGE) with 0.1% Sodium Dodecyl Sulfate 

The presence of fibrils in the samples after two weeks of incubation at 37 °C was confirmed by gel-electrophoresis in 4% PAGE in the presence of 0.1% sodium dodecyl sulfate (SDS). In this case, the samples were incubated with 2% SDS at 37 °C for 5 min and immediately loaded onto a gel [29]. The gels were stained with Coomassie G-250. 

### 4.9. AFM Measurements 

To prepare samples for AFM 2 µL of solution from the protein sample incubated at 37 °C (with a concentration of 0.1 mg/mL) was taken, the solution was transferred to freshly cleaved mica and incubated for 5 min. The sample was then washed three times in a drop of distilled water for 30 seconds and dried in the air. Similarly, all studied samples were prepared, which were subsequently sampled depending on the incubation time and loaded on mica. 

AFM imaging was performed with AFM Integra-Vita microscope (“NT-MDT”, Zelenograd, Russia) in noncontact (tapping) mode in air. The typical scan rate was 1 Hz. Measurements were carried out using cantilevers NSG03 with a resonance frequency of 47–150 kHz and ensured 10 nm tip curvature radius. The processing and presentations of AFM images were performed using Nova software (“NT-MDT”, Zelenograd, Russia) and Gwyddion 2.44 software (Czech Metrology Institute, Brno, Czech Republic). 

### 4.10. Small Angle X-Ray Scattering (SAXS)

SAXS measurements were performed in the small-angle chamber BL-6A at Photon Factory (Tsukuba, Japan). The protein solution in the thermostated cell with mica windows was irradiated by X-rays with a wavelength of 1.503 Å at 23 °C. The distance between the sample and source was 2.35 m. The range of detectable scattering vectors Q was 0.008–0.2 Å^-1^ (Q = 4πsinθ/λ, where λ—wavelength of X-rays, 2θ-the scattering angle). The data were registered by the two-dimensional CCD X-ray detector PILATUS 100K. The shape of particles was estimated from the slope (tgα) of lgI –lgQ dependence, where I—intensity of scattering, and Q—scattering vector [30]. 

### 4.11. The Congo Red Birefringence Assay

For the microscopic analysis the samples of protein, which were incubated at 37 °C and stained with Congo Red for spectroscopic assay, were transferred from cuvette to an Eppendorf and centrifuged at 12,000 to 14,000 rpm to pellet aggregates. The pellet aggregates were then washed with water, resuspended in a small amount of water and placed on a cover glass. After drying for 24 h the sample was analyzed under polarized light at 40× magnification (Olympus light microscope equipped with polarizers and CCD camera).

### 4.12. Molecular Modelling

The molecules of BIF variants were obtained in MODELLER [31]. The modelling schedule included VTFM-optimization procedure and molecular dynamics in vacuo. After that, they were sorted by their normalized DOPE z-score [32] and the best models were selected. The interactions were analyzed in YASARA View [33] using its internal cutoff for hydrogen bonds (6.25 kJ/mol), and user-defined cutoffs for hydrophobic and π–π interactions (5 Å for both). Solvent-accessible surface was built using 1.4 Å probe radius. 

### 4.13. Statistical Analysis 

Experiments were replicated by independently executing the process of protein preparation, their incubation at 37 °C and sample analysis three times.

The reported curves of ThT fluorescence were obtained by averaging results from three individual experiments. The error bars represent the standard deviation.

lgI-lgQ dependences in SAXS data were approximated by linear regression. Determination coefficients (R^2^) ranged from 0.92 to 0.99.

## Appendix A

**Table A1:** The comparative characteristics of proteins aggregates.

Protein	pH	tgα (SAXS), Type of Aggregates	Binding of ThT * (30 Days)	Binding of CR * (30 Days)	Resistance to 2% SDS (30 Days)	Birefringence of CR (30 Days)
BIF	7.2	−2.84 Mainly branched flat associates (14 days) −1.1 Mainly fibrillar structures (30 days)	+	+	+	+
	6.5	“—” *	+	+	+	+
	4.5	−3.42 Mainly branched structures from globular particles	−	−	−	−
K170N	7.2	−2.0 Mainly branched flat associates (30 days)	−	−	−	−
	6.5	−0.92 Rod-like structures (30 days)	−	−	−	−
	4.5	“—”	−	−	−	−
Y187N	7.2	−1.7 Mainly rod-like structures (30 days)	+	+	+	+
	6.5	“—”	+	+	+	+
	4.5	“—”	+	+	+	+
R109N	7.2	“—”	+	+	+	+
	6.5	“—”	+	+	+	+
	4.5	“—”	−	−	−	−
S157Q	7.2	−2.8 Mainly branched flat associates (14 days)	+	+	+	+
	6.5	“—”	−	−	−	−
	4.5	“—”	−	−	−	−
D29Q	7.2	“—”	+	+	+	+
	6.5	“—”	−	−	−	−
	4.5	“—”	−	−	−	−

## References

[1] Solomon A., Weiss D.T., Murphy C.L., Hrncic R., Wall J.S., Schell M. (1998). Light chain-associated amyloid deposits comprised of a novel kappa constant domain. Proc. Natl. Acad. Sci. USA.

[2] Jiang Y., Li H., Zhu L., Zhou J.M., Perrett S. (2004). Amyloid nucleation and hierarchical assembly of Ure2p fibrils. Role of asparagine/glutamine repeat and nonrepeat regions of the prion domains. J. Biol. Chem..

[3] Perutz M.F. (1999). Glutamine repeats and neurodegenerative diseases: Molecular aspects. Trends Biol. Sci..

[4] Haass C., Steiner H. (2001). Protofibrils, the unifying toxic molecule of neurodegenerative disorders?. Nat. Neurosci..

[5] Walsh D.M., Klyubin I., Fadeeva J.V., Cullen W.K., Anwyl R., Wolfe M.S., Rowan M.J., Selkoe D.J. (2002). Naturally secreted oligomers of amyloid beta protein potently inhibit hippocampal long-term potentiation in vivo. Nature..

[6] Timchenko M.A., Timchenko A.A. (2018). Influence of a single point mutation in the constant domain of the Bence-Jones Protein BIF on its aggregation properties. Biochem. (Mosc.).

[7] Sabate R., Rousseau F., Schymkowitz J., Batlle C., Ventura S. (2015). Amyloids or prions? That is the question. Prion.

[8] Nokwe C.N., Hora M., Zacharias M., Yagi H., John C., Reif B., Goto Y., Buchner J. (2015). The Antibody Light-Chain Linker Is Important for Domain Stability and Amyloid Formation. J. Mol. Biol..

[9] Weber B., Hora M., Kazman P., Göbl C., Camilloni C., Reif B., Buchner J. (2018). The Antibody Light-Chain Linker Regulates Domain Orientation and Amyloidogenicity. J. Mol. Biol..

[10] Roussel A., Spinelli S., Déret S., Navaza J., Aucouturier P., Cambillau C. (1999). The structure of an entire noncovalent immunoglobulin kappa light-chain dimer (Bence-Jones protein) reveals a weak and unusual constant domains association. Eur. J. Biochem..

[11] Kabat E.A., Wu T.T., Perry H.M., Gottesman K.S., Foeller C. (1991). Sequences of Proteins of Immunologic Interest. Nat. Inst. Health.

[12] Myatt E.A., Westholm F.A., Weiss D.T., Solomon A., Schiffer M., Stevens F.J. (1994). Pathogenic potential of human monoclonal immunoglobulin light chains: Relationship of in vitro aggregation to in vivo organ deposition. Proc. Natl. Acad. Sci. USA.

[13] Katina N., Timchenko A., Balobanov V., Vasiliev V., Kashparov I., Bychkova V., Kihara H. (2012). Kinetics of mutant apomyoglobin association. Macromol. Symp..

[14] Feigin L.A., Svergun D.A. (1987). Structure Analysis by Small-Angle X-Ray and Neutron Scattering.

[15] Helms L.R., Wetzel R. (1995). Destabilizing loop swaps in the CDRs of an immunoglobulin V_L_ domain. Protein Sci..

[16] Wall J., Schell M., Murphy C., Hrncic R., Stevens F.J., Solomon A. (1999). Thermodynamic instability of human lambda 6 light chains: Correlation with fibrillogenicity. Biochemistry.

[17] Raffen R., Diekman L.J., Szpunar M., Wanschl C., Rokkuludi P.R., Dave R. (1999). Physicochemical consequences of amino acid variations that contribute to fibril formation by immunoglobulin light chains. Protein Sci..

[18] Kim Y.-S., Wall J.S., Meyer J., Murphy C., Randolph T.W., Manning M. (2000). Thermodynamic modulation of light chain amyloid fibril formation. J. Biol. Chem..

[19] Bliznyukov O.P., Kozmin L.D., Vysotskaya L.L., Golenkov A.K., Tishchenko V.M., Samoylovich M.P., Klimovich V.B. (2005). Human immunoglobulin light chains lambda form amyloid fibrils and granular aggregates in solution. Biochemistry.

[20] Stevens F.J., Myatt E.A., Chang C.H., Westholm F.A., Eulitz M., Weiss D.T., Murphy C., Solomon A., Schiffer M. (1995). A molecular model for self-assembly of amyloid fibrils: Immunoglobulin light chains. Biochemistry.

[21] Nowak M. (2004). Immunoglobulin kappa light chain and its amyloidogenic mutants: A molecular dynamics study. Proteins.

[22] Mukherjee S., Pondaven S.P., Jaroniec C.P. (2011). Conformational flexibility of a human immunoglobulin light chain variable domain by relaxation dispersion nuclear magnetic resonance spectroscopy: Implications for protein misfolding and amyloid assembly. Biochemistry.

[23] Wilkins-Stevens P., Raffen R., Hanson D.K., Deng Y.L., Berrios-Hammond M., Westholm F.A., Murphy C., Eulitz M., Wetzel R., Solomon A. (1995). Recombinant immunoglobulin variable domains generated from synthetic genes provide a system for in vitro characterization of light-chain amyloid proteins. Protein Sci..

[24] Maniatis T., Fritisch E.F., Sambrok J. (1982). A Laboratory Manual. Molecular Cloning.

[25] LaemmLi U.K. (1970). Cleavage of structural proteins during the assembly of the head of bacteriophage T4. Nature.

[26] Dubnovitsky A.P., Kravchuk Z.I., Chumanevich A.A., Cozzi A., Arosio P., Martsev S.P. (2000). Expression, refolding, and ferritin-binding activity of the isolated V_L_-domain of monoclonal antibody F11. Biochemistry.

[27] LeVine H. (1993). Thioflavine T interaction with synthetic Alzheimer’s disease beta-amyloid peptides: Detection of amyloid aggregation in solution. Protein Sci..

[28] Klunk W.E., Jacob R.F., Mason R.P. (1999). Quantifying amyloid beta-peptide (Abeta) aggregation using the Congo Red-Abeta (CR-abeta) spectrophotometric assay. Anal. Biochem..

[29] Kushnirov V.V., Alexandrov I.M., Mitkevich O.V., Shkundina I.S., Ter-Avanesyan M.D. (2006). Purification and analysis of prion and amyloid aggregates. Methods.

[30] Glatter O., Kratky O. (1982). Small Angle X-Ray Scattering.

[31] Sali A., Blundell T.L. (1993). Comparative protein modelling by satisfaction of spatial restraints. J. Mol. Biol..

[32] Shen M.Y., Sali A. (2006). Statistical potential for assessment and prediction of protein structures. Protein Sci..

[33] Krieger E., Vriend G. (2014). YASARA View—molecular graphics for all devices—from smartphones to workstations. Bioinformatics.

